# Differential cyclooxygenase expression levels and survival associations in type I and type II ovarian tumors

**DOI:** 10.1186/s13048-018-0389-9

**Published:** 2018-02-27

**Authors:** Alicia Beeghly-Fadiel, Andrew J. Wilson, Spencer Keene, Meral El Ramahi, Shu Xu, Lawrence J. Marnett, Oluwole Fadare, Marta A. Crispens, Dineo Khabele

**Affiliations:** 1 Department of Medicine, Division of Epidemiology, Vanderbilt Epidemiology Center, Institute for Medicine and Public Health, Vanderbilt University Medical Center, Nashville, TN, USA; 2 Vanderbilt-Ingram Cancer Center, Nashville, TN USA; 3 Department of Obstetrics & Gynecology, Division of Gynecologic Oncology, Vanderbilt University Medical Center, Nashville, TN USA; 4 Department of Biochemistry, Vanderbilt University Medical Center, Nashville, TN USA; 5 Vanderbilt Institute of Chemical Biology, Vanderbilt University Medical Center, Nashville, TN USA; 6 Department of Pathology, School of Medicine, University of California, San Diego, La Jolla, CA USA; 7 Department of Obstetrics and Gynecology, Division of Gynecologic Oncology, The University of Kansas Medical Center, MS 2028, 3901 Rainbow Boulevard, Kansas City, KS 66160 USA

**Keywords:** Cyclooxygenase, Ovarian cancer, Tumor subtype, Survival analysis

## Abstract

**Background:**

High cyclooxygenase (COX)-2 expression in ovarian tumors has been associated with poor prognosis, but the role of COX-1 expression and its relation to survival is less clear. Here, we evaluated COX expression and associations with survival outcomes between type I (clear cell, mucinous, low grade endometrioid and low grade serous) and type II (high grade serous and high grade endometrioid) ovarian tumors.

**Methods:**

We developed and validated a new COX-1 antibody, and conducted immunohistochemical (IHC) staining for COX-1 and COX-2 on a tissue microarray (TMA) of 190 primary ovarian tumors. In addition to standard IHC scoring and H-scores to combine the percentage of positive cells and staining intensity, we also measured COX-1 and COX-2 mRNA expression by QPCR. High expression was defined as greater than or equal to median values. Clinical characteristics and disease outcomes were ascertained from medical records. Associations with disease-free survival (DFS) and overall survival (OS) were quantified by hazard ratios (HRs) and confidence intervals (CIs) from proportional hazards regression.

**Results:**

Type I tumors had high COX-2 expression, while type II tumors had high COX-1 expression. In multivariable adjusted regression models, higher COX-1 mRNA expression was associated with shorter DFS (HR: 6.37, 95% CI: 1.84–22.01) and OS (HR: 2.26, 95% CI: 1.04–4.91), while higher H-scores for COX-2 expression were associated with shorter DFS (HR: 1.92, 95% CI: 1.06–3.49). Stratified analysis indicated that COX-2 was significantly associated with DFS among cases with Type II tumors (HR: 1.93, 95% CI: 1.06–3.53).

**Conclusions:**

These findings suggest that ovarian tumor type contributes to differences in COX expression levels and associations with survival.

**Electronic supplementary material:**

The online version of this article (10.1186/s13048-018-0389-9) contains supplementary material, which is available to authorized users.

## Background

Ovarian cancer is the 5th leading cause of cancer deaths among women in the United States (US), and has an overall five year survival rate of only 46% [1]. Surgical tumor debulking and a combination of platinum and taxane chemotherapy are standard treatment for ovarian cancer [2]. While survival rates have modestly improved over time, the mortality rate of ovarian cancer has not substantially changed for nearly 50 years, remaining at approximately 10 women per 100,000 [2–4]. Identifying additional ways to improve treatment, extend survival, and decrease mortality are critical research priorities for ovarian cancer [2, 3].

Ovarian cancer is classified into two distinct types with different etiologies based on histology and grade: indolent type I and aggressive type II tumors [2, 5, 6]. Clear cell, mucinous, low grade endometrioid and low grade serous cancers are type I, whereas high grade serous, high grade endometrioid, carcinosarcomas, and undifferentiated cancers are type II tumors [2, 5]. Type I tumors typically arise from benign precursor lesions and are generally slow-growing, low grade, and chromosomally stable. In contrast, type II tumors are fast-growing, high grade, and genetically unstable [2, 5]. In addition to differences in etiology, prognosis differs, as women with type I tumors generally fare much better than those with type II tumors [2, 7].

Two cyclooxygenase (COX) isoforms, also known as prostaglandin H_2_ synthases (PTGS or PGHS), are rate-limiting enzymes that produce prostaglandins by catalyzing the oxidation of arachidonic acid to tissue-specific lipid prostanoids [8, 9]. Effects downstream of COX include inflammation, platelet activation, cellular proliferation, angiogenesis, invasion, and metastasis [9, 10]. COX-2 is strongly implicated in ovarian cancer progression; meta-analysis of 15 studies found 35% significantly worse survival for cases with COX-2 positive tumors [11]. Despite this evidence, questions remain, as studies included in the meta-analysis were small (average *N* = 104), included predominantly non-US study populations, and had significant heterogeneity across findings [11]. COX-1 may also be relevant to ovarian cancer progression. Unlike most other solid malignancies where COX-2 expression is high, our group and others have found higher COX-1 than COX-2 levels in high grade serous ovarian cancer [12–14]. Despite this unique expression, only a few small studies have evaluated COX-1 in ovarian cancer survival, and evidence of an association has been limited [15–17]. In one small study, semi-quantitative PCR indicated that serous and entrometrioid had higher, while clear cell carcinomas had lower COX-2 levels [18]. Other studies that used immunohistochemistry (IHC) found that both COX-1 and COX-2 were higher in non-mucinous than mucinous tumors [16, 19]. Our prior report showed higher COX-1 than COX-2 in serous tumors [12]. One study of 65 cases found no difference in COX-2 expression by tumor type [20], but both COX isozymes were significantly associated with tumor type in another studyof 82 type I and 131 type II tumors [21]. Analyses stratified by tumor type are limited with regard to survival, and no prior reports have evaluated the significance of an interaction term between COX expression and tumor type in multivariable regression models.

The purpose of this study was to determine if COX expression and associations with survival outcomes differ between type I and type II ovarian tumors. Since possible cross-reactivity between IHC antibodies for COX-1 and COX-2 may have influenced prior findings, we generated and validated a new COX-1 antibody. We then measured COX-1 and COX-2 expression in type and type II ovarian tumors and tested associations with disease-free survival (DFS) and overall survival (OS) from ovarian cancer. These findings may have important implications for disease prognosis and for COX targeted therapeutic interventions for women with ovarian cancer.

## Methods

### Antibody generation

Generation of a new rabbit polyclonal anti-COX-1 antibody was conducted at the Vanderbilt Antibody and Protein Resource. The protein sequence of human COX-1 and COX-2 were compared to identify unique COX-1 sequences [22]. Anti–human COX-1 antibodies were generated by immunization of two New Zealand white rabbits with three KLH-conjugated human COX-1 specific peptides (peptide sequences: CQDDGPAVERPS, ADPGAPTPVC, and LMHYPRGIPPQSQMAC) which have no overlap with the corresponding COX-2 protein sequence (Additional file 1). Bleeds from sequential boosts were collected from both rabbits and tested by Western blot and ELISA. Antisera from both animals were mixed for affinity purification. Polyclonal antibodies were purified using affinity against immunizing peptides and then the elution was sequentially cleared by a mouse COX-2 immobilized affinity column. Final antibodies were further validated by Western blot, and demonstrated to have no cross-reactivity with COX-2 (Additional file 1). Antibodies used for Western blot included the newly generated Vanderbilt rabbit polyclonal anti-COX-1 (1:2000 overnight), rabbit polyclonal anti-COX-2 (1:1000 overnight; Cayman Chemicals, Ann Arbor, MI), and mouse monoclonal anti-actin (1:10000 for 15 mins; Sigma Chemical Co, St Louis, MO); positive controls included recombinant ovine COX-1 and human COX-2 (both from Cayman Chemicals).

### Laboratory analysis

A tissue microarray (TMA) was created from primary ovarian cancer samples from the Vanderbilt Tissue Repository for Ovarian Cancer (TROC), as previously described [12]. We conducted immunostaining with the newly generated rabbit polyclonal COX-1 antibody (designated Vanderbilt), the well-characterized commercially available mouse monoclonal COX-1 antibody (Cat#sc-19,998; Santa Cruz Biotechnology, Dallas, TX), and a widely-used mouse monoclonal COX-2 antibody l (Cat# 18–7379; Thermo Fisher Scientific, Inc., Waltham, MA), as previously described [12]. Immunohistochemistry (IHC) was performed in the Vanderbilt Translational Pathology Shared Resource. Whole-slide imaging and semi-quantitative measurement of the percentage of tumor cells showing positive cytoplasmic expression was performed using the automated Ariol® SL-50 Platform in the Digital Histology Shared Resource (DHSR) of the Vanderbilt University Medical Center (VUMC). For comparability with existing literature, IHC staining was first categorized as weak (< 10% positive), moderate (10–50% positive), or strong (> 50% positive). In addition to this standard IHC scoring, we also multiplied the the percentage of cells staining positive (0–100) by staining intensity (weak: 1, moderate: 2, strong: 3), which resulted in an H-score ranging from 0 to 300 [23]. Steady-state expression levels of COX-1 and COX-2 were also measured at the mRNA level by quantitative real-time PCR (QPCR), as previously described [12].

### Statistical analysis

Correlations between continuous variables were evaluated with Pearson’s correlation coefficient. Standard IHC scoring was dichotomized as weak or moderate/strong; expression measured by H-scores and QPCR was dichotomized, with high defined as greater than or equal to median values. Associations with COX expression were assessed with Student’s t-tests for continuous or *x*^2^ tests for categorical variables. Overall survival (OS) was defined as the interval between cancer diagnosis and death, or else censored at the date of last contact in medical records. Disease-free survival (DFS) was defined as the interval between cancer diagnosis and evidence of disease recurrence, or else censored at the date of death or last contact; women that died without a disease-free interval were excluded from these analyses. Associations with survival were evaluated using Cox proportional hazards regression to calculate hazard ratios (HR) and corresponding 95% confidence intervals (CI). Clinical covariates of interest included age, stage of disease, histologic subtype, grade, residual disease, and platinum sensitivity. High grade serous and high grade endometrioid tumors were classified as type II tumors; all others were classified as type I tumors [24]. Differences in associations by tumor type were evaluated in stratified models; to formally test statistical interactions, multiplicative terms were created and included in regression models. Kaplan-Meier functions were employed to visually evaluate survival outcomes; differences in curves were evaluated with Log Rank tests. A *P*-value ≤ 0.05 was interpreted as statistically significant. All analyses were conducted with SAS v 9.4.

## Results

**Fig. 1:**
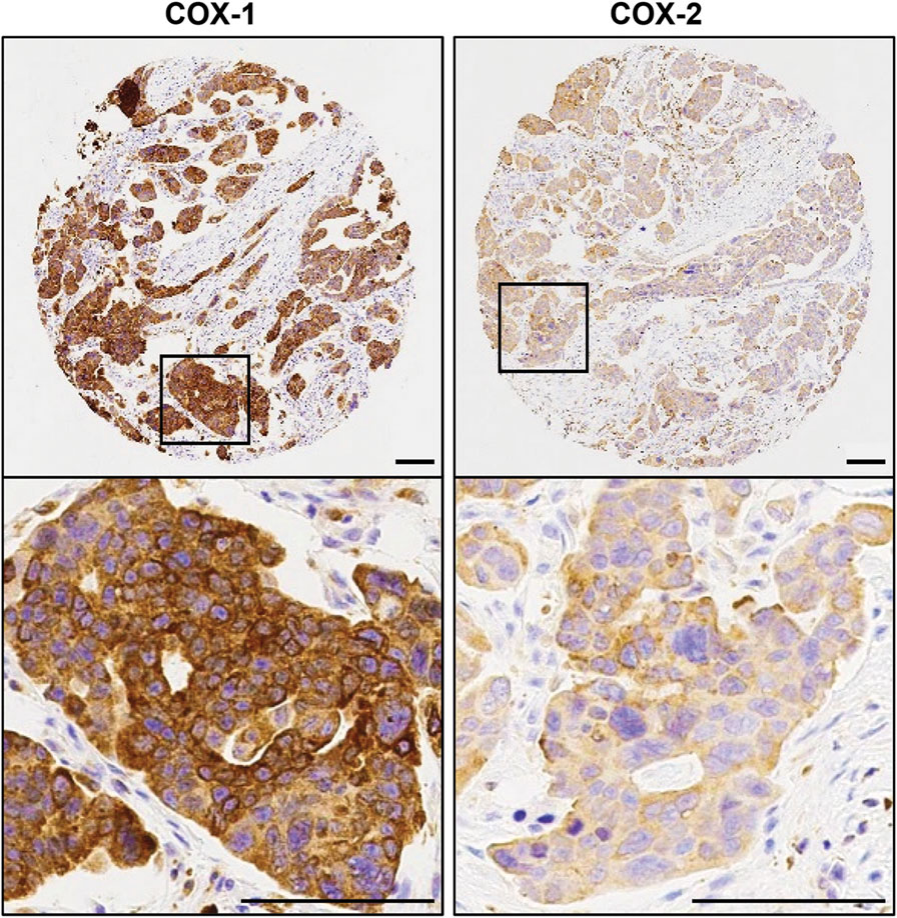
Representative IHC Images for COX-1 and COX-2, the Vanderbilt TROC. Representative immunohistochemical staining for COX-1 and COX-2 in a high-grade serous (VOC-A-155) section from a TMA of 190 ovarian tumors. Higher power images of the boxed areas are shown in the corresponding right panels. Scale bars represent 100 μM

**Fig. 2:**
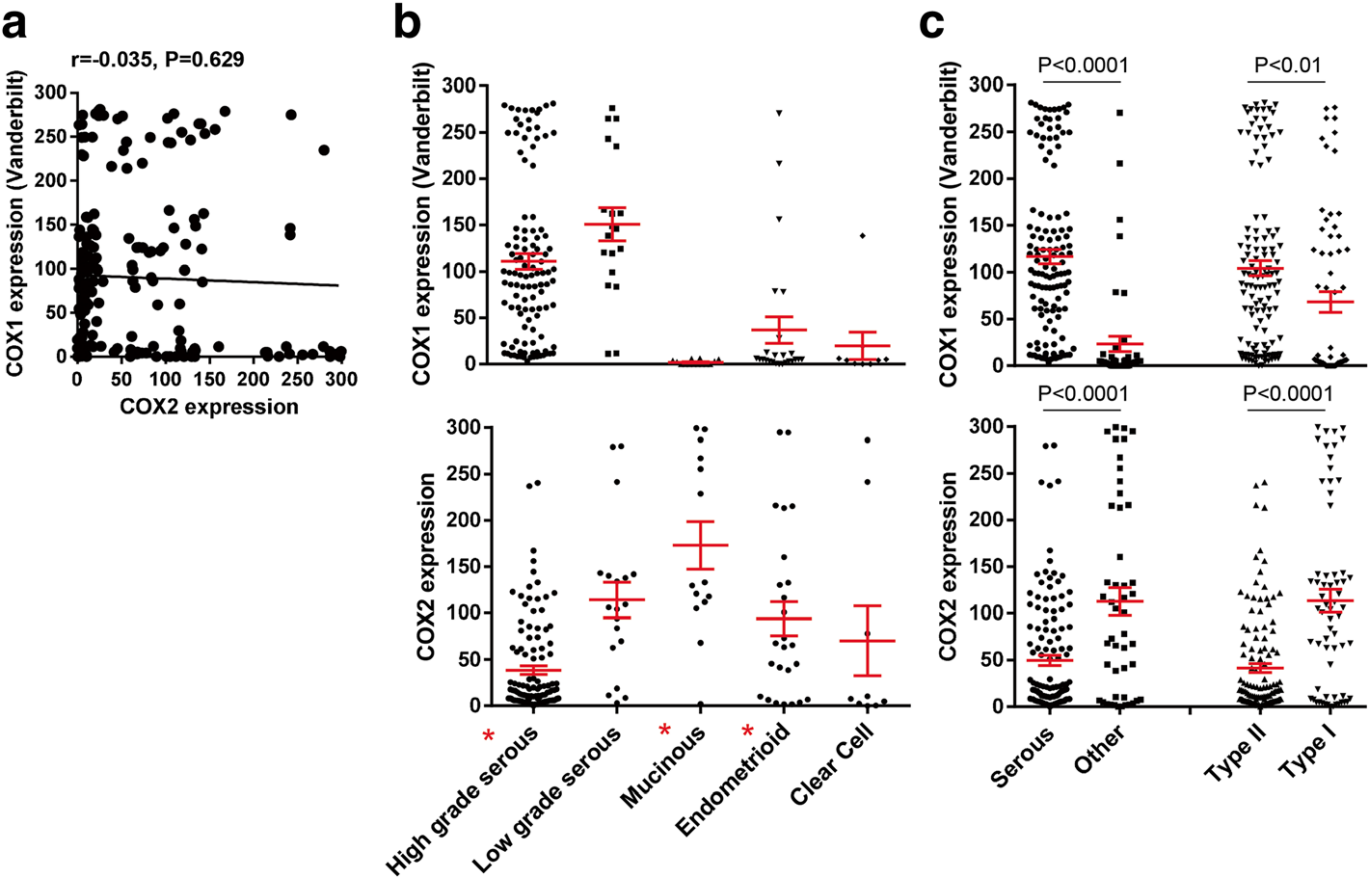
COX-1 and COX-2 protein expression in ovarian cancer. The percentage of tumor cells positive for COX-1 and COX-2 was determined by automated image analysis and then scaled by intensity for H-scores, ranging from 0 to 300. **a** Pearson correlation between expression levels of COX-1 and COX-2. H-scores for COX-1 and COX-2 expression in (**b**) serous, endometrioid, mucinous and clear cell tumors, and in (**c**) serous tumors versus all other epithelial tumors and type II tumors (high grade serous and high grade endometrioid) versus type I tumors (all other types). *P*-values from Student’s t-tests

A total of 190 epithelial ovarian cancer cases from the Vanderbilt TROC were evaluated for COX expression by IHC; representative staining images in a high-grade serous tumor are shown in Fig. 1. The majority of cases evaluated were Caucasian (88.4%), and had high grade (82.6%), late stage (III or IV = 69.0%), and serous histology (60.0%; Table 1). COX-1 H-scores for the new Vanderbilt and Santa Cruz commercial antibody were 97.6% correlated, and were not related to COX-2 expression levels by IHC (Fig. 2a & Additional file 2). Unless otherwise stated, COX-1 IHC expression data was hereafter from the new Vanderbilt developed antibody.

**Table 1:** Clinical characteristics of 190 epithelial ovarian cancer cases from the Vanderbilt TROC

Characteristic	N or mean	% or std. dev
Age at Diagnosis, years	57.9	(13.7)
Overall Survival, years	4.3	(3.4)
Disease-Free Survival, years	3.9	(3.4)
Race		
White	168	(88.4)
African American	11	(5.8)
Other/Unknown	11	(5.8)
Histologic Subtype		
Serous	131	(69.0)
Endometrioid	25	(13.2)
Mucinous	14	(7.4)
Clear Cell	9	(4.7)
Mixed	6	(3.2)
Other	5	(2.6)
Stage		
I	50	(26.3)
II	7	(3.7)
III	110	(57.9)
IV	21	(11.1)
Unstaged	2	(1.1)
Grade		
Low Grade	33	(17.4)
High Grade	157	(82.6)
Tumor Type		
Type I	65	(34.2)
Type II	125	(65.8)
Residual Disease		
Optimal Debulking	47	(24.7)
Suboptimal Debulking	89	(46.8)
Unknown or Not Applicable	54	(28.4)
Platinum Sensitive Disease		
Resistant	39	(20.5)
Sensitive	81	(42.6)
Unknown or Not Applicable	70	(36.8)

Percentages may not sum to 100 due to rounding error

Expression of both COX isozymes, as measured by H-score, significantly differed in high grade serous, mucinous, and endometrioid ovarian cancers (Fig. 2b). High COX-1 levels were observed in both low and high grade serous tumors, and were significantly lower in tumors of non-serous histology. Biologically aggressive Type II tumors (high grade serous and high grade endometrioid) had significantly higher COX-1 expression than Type I tumors. A similar pattern of COX-1 expression was observed with the commercial antibody (Additional file 2). In contrast, COX-2 levels were significantly higher in non-serous and type I tumors (Fig. 2b and c). To further validate the specificity of our antibodies, we also measured COX gene expression by QPCR. Among 49 serous cases with both COX-1 measures available, protein and mRNA levels were highly correlated for both the newly developed Vanderbilt COX-1 antibody (*r* = 0.764, *P* < 0.0001) and the commercial COX-1 antibody (*r* = 0.763, *P* < 0.0001); similarly, for 36 serous cases with both COX-2 measures available, H-scores and mRNA measures were highly correlated (*r* = 0.738, *P* < 0.0001) (Additional file 3).

Associations between COX IHC and clinical covariates are shown in Table 2. Regardless of whether standard IHC or H-Scores were evaluated, cases with high COX-1 expression levels were more likely to have late stage disease, serous histology, and to be type II tumors (all *P*-values < 0.05). For COX-2, cases with high expression levels were more likely to have early stage disease, non-serous histology, less differentiation, optimal debulking, platinum sensitivity, and be type I tumors (all *P*-values < 0.05). High COX-2 was also associated with younger age at diagnosis, but only when measured by H-score.

**Table 2:** Associations between COX expression levels and clinical covariates, the Vanderbilt TROC

	COX-1 / PTGS-1					COX-2 / PTGS-2				
	Standard IHC scoring			H-Score		Standard IHC scoring			H-Score	
Characteristic	N (%) weak	Moderate & strong	*P*-value	Mean (std err)	*P*-value	N (%) weak	Moderate & strong	*P*-value	Mean (std err)	*P*-value
Age										
< 57.6	39 (50.7)	56 (49.6)	0.883	91.2 (9.4)	0.889	46 (44.7)	49 (56.3)	0.109	77.6 (8.8)	**0.047**
≥ 57.6	38 (49.4)	57 (50.4)		93.0 (9.3)		57 (55.3)	38 (43.7)		54.6 (7.3)	
Stage										
I/II	32 (41.6)	25 (22.1)	**0.004**	72.1 (11.9)	**0.047**	17 (16.5)	40 (46.0)	**< 0.001**	111.5 (12.8)	**< 0.001**
III/IV/Unstaged	45 (58.4)	88 (77.9)		100.6 (7.9)		86 (83.5)	47 (54.0)		46.7 (5.4)	
Histologic Subtype										
Serous	47 (61.0)	12 (10.6)	**< 0.001**	116.5 (7.6)	**< 0.001**	82 (79.6)	49 (56.3)	**< 0.001**	49.7 (5.4)	**< 0.001**
Non-Serous	30 (39.0)	101 (89.4)		37.9 (9.7)		21 (20.4)	38 (43.7)		102.7 (13.2)	
Grade										
Low Grade	15 (19.5)	18 (15.9)	0.526	92.4 (16.4)	0.982	5 (4.9)	28 (32.2)	**< 0.001**	134.0 (16.1)	**< 0.001**
High Grade	62 (80.5)	95 (84.1)		92.0 (7.2)		98 (95.2)	59 (67.8)		51.9 (5.5)	
Type										
Type I Tumors	39 (50.7)	26 (23.0)	**< 0.001**	68.4 (11.1)	**0.009**	19 (18.5)	46 (52.9)	**< 0.001**	113.4 (12.2)	**< 0.001**
Type II Tumors	38 (49.4)	87 (77.0)		104.4 (8.0)		84 (81.6)	41 (47.1)		41.5 (4.8)	
Residual Disease^a^										
Optimal	22 (43.1)	25 (29.4)	0.103	78.4 (12.2)	0.177	16 (21.1)	31 (51.7)	**< 0.001**	109.1 (14.0)	**< 0.001**
Suboptimal	29 (56.9)	60 (70.6)		97.3 (9.3)		60 (79.0)	29 (48.3)		42.4 (6.1)	
Platinum Sensitivity^b^										
Resistant	12 (30.0)	27 (33.8)	0.679	93.0 (13.6)	0.884	28 (40.0)	11 (22.0)	**0.038**	37.7 (8.7)	**0.030**
Sensitive	28 (70.0)	53 (66.3)		95.5 (9.7)		42 (60.0)	39 (78.0)		63.8 (8.2)	

Bold values denote signficant associations; column percentages may not sum to 100% due to rounding error

^a^Among cases with known cytoreductive status

^b^Among cases treated with platinum with response to treatment known

Next, we evaluated COX expression in relation to ovarian cancer survival (Table 3). By standard IHC scoring, high COX-1 was associated with significantly worse DFS in unadjusted analysis (HR: 2.34, 95% CI: 1.30–4.24), but this association was attenuated after multivariable adjustment. On the contrary, COX-1 expression by QPCR was associated with a significantly shorter disease-free interval after both minimal (HR: 4.12, 95% CI: 1.51–11.26) and full multivariable adjustment (HR: 6.37, 95% CI: 1.84–22.01), and significantly shorter OS after multivariable adjustment (HR: 2.26, 95% CI: 1.04–4.91). In crude analyses, high COX-2 was associated with significantly better DFS (HR: 0.51, 95% CI: 0.30–0.87) and OS (HR: 0.52, 95% CI: 0.36–0.77) by standard IHC, and significantly better OS by H-score (HR: 0.57, 95% CI: 0.39–0.82). However, these associations did not withstand adjustment for clinical covariates, and instead high COX-2 by H-score was associated with significantly shorter DFS in both minimally (HR: 1.93, 95% CI: 1.08–3.46) and fully adjusted models (HR: 1.92, 95% CI: 1.06–3.49).

**Table 3:** Associations between COX expression levels and ovarian cancer survival, the Vanderbilt TROC

	Disease-free survival (DFS), hazard ratio (95% CI), *P*-value						Overall survival (OS), hazard ratio (95% CI), *P*-value					
	IHC^1^		H-Score^2^		QPCR^3^		IHC^1^		H-Score^2^		QPCR^3^	
COX-1 / PTGS1												
N cases / events^a^	47 / 15 vs 71 / 42		60 / 26 vs 58 / 31		15 / 12 vs 17 / 13		77 / 43 vs 113 / 77		95 / 57 vs 95 / 63		24 / 19 vs 25 / 18	
Unadjusted	**2.34 (1.30–4.24)**	**0.005**	1.49 (0.88–2.50)	0.138	1.77 (0.79–3.98)	0.168	1.13 (0.78–1.65)	0.515	1.09 (0.76–1.56)	0.647	1.17 (0.61–2.23)	0.641
Adjusted^b^	1.58 (0.85–2.94)	0.148	1.44 (0.85–2.43)	0.180	**4.12 (1.51–11.26)**	**0.006**	0.79 (0.54–1.16)	0.227	0.97 (0.68–1.40)	0.882	2.02 (0.96–4.22)	0.063
Adjusted^c^	1.55 (0.81–2.98)	0.187	1.30 (0.75–2.34)	0.356	**6.37 (1.84–22.01)**	**0.003**	0.87 (0.59–1.30)	0.495	1.14 (0.78–1.65)	0.504	**2.26 (1.04–4.91)**	**0.039**
COX-2 / PTGS2												
N cases / events	50 / 33 vs 68 / 24		46 / 29 vs 72 / 28		10 / 10 vs 14 / 9		103 / 80 vs 87 / 40		95 / 72 vs 95 / 48		18 / 16 vs 18 / 11	
Unadjusted	**0.51 (0.30–0.87)**	**0.013**	0.61 (0.36–1.02)	0.061	0.51 (0.20–1.29)	0.154	**0.52 (0.36–0.77)**	**< 0.001**	**0.57 (0.39–0.82)**	**0.002**	0.59 (0.27–1.27)	0.173
Adjusted^b^	1.53 (0.85–2.76)	0.160	**1.93 (1.08–3.46)**	**0.028**	0.86 (0.30–2.45)	0.772	0.85 (0.57–1.27)	0.420	0.89 (0.61–1.31)	0.559	0.77 (0.35–1.71)	0.519
Adjusted^c^	1.54 (0.84–2.80)	0.161	**1.92 (1.06–3.49)**	**0.033**	0.87 (0.31–2.47)	0.798	1.05 (0.69–1.59)	0.818	1.05 (0.71–1.56)	0.802	1.40 (0.56–3.51)	0.477

^1^COX protein measured by immunohistochemistry: moderate & strong vs weak

^2^COX protein measured by H-Score: dichotomized at median

^3^COX gene expression measured by quantitative real-time PCR: dichotomized at median

^a^N cases / N events, reference vs high COX

^b^Adjusted for age at diagnosis (continuous), stage (early, late), and tumor type (I, II)

^c^Additionally adjusted for debulking (optimal, suboptimal, missing) and platinum sensitivity (sensitive, resistant, missing) Bold values denote signficant associations; column percentages may not sum to 100% due to rounding error

**Fig. 3:**
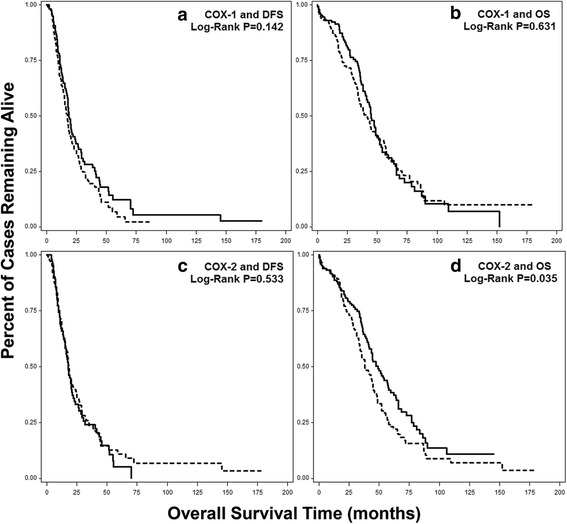
Kaplan-Meier Survival Functions for COX-1 and COX-2 by RNA-seq from TCGA. Normalized RNA-seq data downloaded from the Broad Firehose for 489 high grade serous (type II) cases, analyzed for (**a**) COX-1 and DFS, (**b**) COX-1 and OS, (**c**) COX-2 and DFS, and (**d**) COX-2 and OS. Solid line = lower than median COX expression; dotted line = median or higher COX expression. X-axis is overall survival time in months, Y-axis is percentage of cases remaining alive, *P*-values from Log-Rank tests

To further clarify the role of COX expression in ovarian cancer survival, we also evaluated data for 489 high grade serous ovarian cancer cases from The Cancer Genome Atlas (TCGA). We downloaded normalized RNA-seq mRNA data and survival times from the Broad Institute Firehose (https://gdac.broadinstitute.org) and found that cases with median or higher COX-1 levels tended to have shorter DFS (HR: 1.25, 95% CI: 0.93–1.68), but no association with OS (HR: 1.07, 95% CI: 0.80–1.44). When we applied this approach to COX-2, no association was found for DFS (HR: 0.91, 95% CI: 0.68–1.27), but high COX-2 was significantly associated with worse OS (HR: 1.36, 95% CI: 1.02–1.82). As expected, Kaplan-Meier survival functions (Fig. 3) were found to be in agreement with unadjusted analyses, with a suggestive association for high COX-1 and DFS (*P*-value = 0.142) and significantly worse OS for high COX-2 (*P*-value = 0.035).

Because TCGA includes only high grade serous ovarian cancers, interactions with tumor type could not be evaluated in this data. Similarly, nearly all cases for which we conducted QPCR were type II tumors. Thus, to determine if associations differed between type I and type II tumors, we evalutated dichotomized COX H-scores in stratified analyses (Table 4). High COX-2 was associated with better OS in unadjusted analysis among type I cases (HR: 0.38, 95% CI: 0.16–0.88), and worse DFS in multivariable adjusted analyses among type II cases (HR: 1.93, 95% HR: 1.06–3.53). While these associations were significant within strata, they did not significantly differ across strata, as statistical interaction terms between COX-2 and tumor type were only suggestive for DFS (P-interaction = 0.181) and OS (P-interaction = 0.051).

**Table 4:** Associations between COX H-score and ovarian cancer survival by tumor type, the Vanderbilt TROC

	Disease-free survival			Overall survival		
	HR (95% CI), *P*-value		P-interaction	HR (95% CI), *P*-value		P-interaction
COX-1 / PTGS1						
Among Type I Cases (*N* = 65)						
N cases / events^a^	28 / 3 vs 21 / 5			39 / 14 vs 26 / 8		
Unadjusted	2.20 (0.52–9.20)	0.282	0.537	0.71 (0.29–1.70)	0.435	0.210
Adjusted^b^	2.88 (0.55–15.04)	0.209		0.56 (0.23–1.38)	0.210	
Among Type II Cases (*N* = 125)						
N cases / events^a^	32 / 23 vs 37 / 26			56 / 43 vs 69 / 55		
Unadjusted	1.32 (0.75–2.31)	0.339		1.10 (0.74–1.64)	0.636	
Adjusted^b^	1.34 (0.76–2.37)	0.313		1.07 (0.72–1.60)	0.740	
COX-2 / PTGS2						
Among Type I Cases (*N* = 65)						
N cases / events^a^	10 / 2 vs 39 / 6			19 / 11 vs 46 / 11		
Unadjusted	0.90 (0.18–4.52)	0.899	0.181	**0.38 (0.16–0.88)**	**0.023**	0.051
Adjusted^b^	0.51 (0.08–3.40)	0.490		0.49 (0.20–1.15)	0.102	
Among Type II Cases (*N* = 125)						
N cases / events^a^	36 / 27 vs 33 / 22			76 / 61 vs 49 / 37		
Unadjusted	1.07 (0.60–1.88)	0.829		0.84 (0.56–1.27)	0.413	
Adjusted^b^	**1.93 (1.06–3.53)**	**0.033**		1.08 (0.71–1.63)	0.723	

^a^N cases / N events, reference vs high COX, measured by H-score, dichotomized at the median

^b^Adjusted for age at diagnosis (continuous) and stage (early, late) Bold values denote signficant associations; column percentages may not sum to 100% due to rounding error

## Discussion

To test our hypothesis that COX expression levels differ across type I and type II ovarian tumors, we measured both COX-1 and COX-2 isozymes in samples from 190 primary ovarian cancer cases from the Vanderbilt TROC. Because cross-reactivity between COX-1 and COX-2 may have influenced prior findings, we developed and validated a new rabbit COX-1 polyclonal antibody. Our new COX-1 antibody had no cross-reactivity with COX-2 and excellent correlation with the commercial COX-1 antibody that we previously evaluated [12]. Our assays were both sensitive and specific as indicated by significant correlations between protein and mRNA expression for both COX-1 and COX-2, and no relationship between measures across the two isoforms. For protein expression by IHC, we also compared standard scoring and H-scores that incorporated both the percentage of positive tumor cells and the staining intensity. Regardless of assay, our analysis indicates that COX-1 and COX-2 expression levels differ across ovarian tumor types: high COX-1 expression was found in type II tumors and high COX-2 expression was found in type I tumors.

With regard to patient outcomes, high COX-1 was associated with shorter DFS in unadjusted models when measured by IHC and in models adjusted for clinical covariates when measured by QPCR. On the contrary, COX-2 measured by QPCR was not associated with any outcome, while high COX-2 by IHC was associated with shorter DFS after adjustment for clinical covariates. RNA-seq data from TCGA also suggested an association between high COX-1 and worse DFS, and showed a significant association for high COX-2 and worse OS. Thus, methodological differences, such as measurement of mRNA or protein, adjustment for clinical covariates, and heterogeneity of tumor types contribute to variation in associations between COX expression and ovarian cancer survival.

Numerous prior reports on COX-2 support a relation with ovarian cancer survival [15, 18, 19, 21, 25–28], and meta-analysis of 15 studies yielded a significantassociation with OS (HR: 1.34, 95% CI: 1.05–1.71) [11]. However, results across studies were highly heterogeneous (I^2^ = 56.5%) [11]. Based on current findings, possible reasons underlying the wide variation across prior reports includes differences in statistical methods, such as covariates adjusted for in regression models, and laboratory differences, such as assays and thresholds used to define positive expression. With regard to DFS, our findings from standard IHC and H-scores did show some variation, with HRs of 1.54 and 1.92, respectively. These generally agreewith findings from meta-analysis of 5 studies (HR: 1.36, 95% CI: 0.79–2.33) [11]. However, our results also suggest that COX-2 OS associations may vary by tumor type, with increased hazards only among type II tumors. Prior studies that stratified by tumor type are limited. In one analysis of 23 type I and 42 type II ovarian cancers, high COX-2 expression was not significantly associated with survival [20]. However, in a larger study where COX-2 was not associated with OS among all cases, significantly worse survival was found when analyses were restricted to 131 type II tumors [21]. Thus, studies that include both type I and type II tumors without conducting stratified analysis may also contribute to the high heterogeneity of results in the existing COX-2 literature.

Our group and others have previously shown that COX-1 is highly expressed in ovarian cancer [12–14]. However, prior studies on COX-1 and ovarian cancer survival are limited. Among 30 optimally debulked serous cases, patients whose tumors had high COX-1 tended to have worse OS (HR: 2.5, 95% CI: 0.81–7.95) [17]. Among 70 and 107 cases, Kaplan-Meier plots for COX-1 were not significant, but proportional hazards regression was not conducted [16, 29]. Two more studies also reported no association, but showed no COX-1 results among 75 and 213 cases [15, 21]. In the current study, analysis of TCGA data was suggestive, and COX-1 was significantly associatedwith shorter DFS and OS when measured by QPCR. As our previous findings indicatethat COX-1 is associated with multiple pro-tumorigenic pathways in high grade serous ovarian cancer [12], more research is needed to determine if COX-1 has utility as a prognostic biomarker for type II serous ovarian cancer.

Strengths of the current study include measuring both COX isozymes by QPCR and IHC, and evaluating both standard IHC and H-scores. Our rigorous statistical analysis included multivariable adjusted regression models, stratified analyses, and interactions with tumor type. We found that tumor type and adjustment for clinical covariates markedly influenced many results, so analyses that do not consider these important factors may have inaccurate results. The primary limitation of this study is our small sample size, such that precision, and power to detect associations, especially in stratified analyses, may be low. However, our TMA is larger than what was included in many prior reports on COX expression and ovarian cancer survival.

## Conclusions

In conclusion, this study indicates that COX-1 expression is high in type II tumors, COX-2 expression is high in type I tumors, and that both COX-1 and COX-2 may influence ovarian cancer outcomes, with possible variation by tumor type. These findings provide strong rationale for additional research that includes larger study populations and methods to disentangle the roles of the two COX isozymes in type I and type II ovarian tumors, which may impact disease prognosis and COX targeted therapeutic interventions for women with ovarian cancer.

## Additional files

Additional file 1: Characterization of new rabbit polyclonal anti-COX-1. **A)** Protein sequences of human COX-1 and COX-2 show that peptides used in anti-COX-1 generation (shaded in yellow) had no overlap with the corresponding COX-2 sequence. **B)** Western blot analysis using the newly generated Vanderbilt anti-COX-1 (1:2000 overnight) show detection of COX-1 at the expected molecular weight (approximately 68 kDa). COX-2 was detected by rabbit polyclonal anti-COX-2. Actin was used as loading control. Samples used were COX-1-expressing OVCAR-3 cell lysate, COX-2-expressing 4 T1 cell lysate, recombinant ovine COX-1 and recombinant human COX-2. (PPTX 71 kb)

Additional file 2: COX-1 expression in ovarian cancer measured by the commercial Santa Cruz antibody (IHC). **A)** Pearson correlation between IHC expression levels of COX-1 and COX-2. H-scores for COX-1 expression in **B)** serous, endometrioid, mucinous and clear cell tumors, and in **C)** serous tumors versus all other epithelial tumors, and type II versus type I tumors. *P* values were determined by Student’s t-test. (PPTX 377 kb)

Additional file 3: COX Expression: Correlation between QPCR and IHC. Pearson correlations between COX expression levels measured by quantitative PCR and H-scores from IHC. (PPTX 235 kb)

## Abbreviations

CI: Confidence interval
COX: Cyclooxygenase
DFS: Disease-free survival
DHSR: Digital Histology Shared Resource
HR: Hazard ratio
IHC: Immunohistochemistry
OS: Overall survival
PTGS or PGHS: Prostaglandin H_2_ synthases
QPCR: Quantitative real-time RT PCR
TCGA: The Cancer Genome Atlas
TMA: Tissue microarray
TROC: Tissue repository for ovarian cancer
VUMC: Vanderbilt University Medical Center
