# Supplementary material for: Differential cyclooxygenase expression levels and survival associations in type I and type II ovarian tumors
Source: J Ovarian Res. 2018 Feb 27;11:17. doi: 10.1186/s13048-018-0389-9 (PMC5828488; doi:10.1186/s13048-018-0389-9)
Supplement: Supplementary file 1 — Characterization of new rabbit polyclonal anti-COX-1. A) Protein sequences of human COX-1 and COX-2 show that peptides used in anti-COX-1 generation (shaded in yellow) had no overlap with the corresponding COX-2 sequence. B) Western blot analysis using the newly generated Vanderbilt anti-COX-1 (1:2000 overnight) show detection of COX-1 at the expected molecular weight (approximately 68 kDa). COX-2 was detected by rabbit polyclonal anti-COX-2. Actin was used as loading control. Samples used were COX-1-expressing OVCAR-3 cell lysate, COX-2-expressing 4 T1 cell lysate, recombinant ovine COX-1 and recombinant human COX-2. (PPTX 71 kb) [file 13048_2018_389_MOESM1_ESM.pptx]

## Slide 1
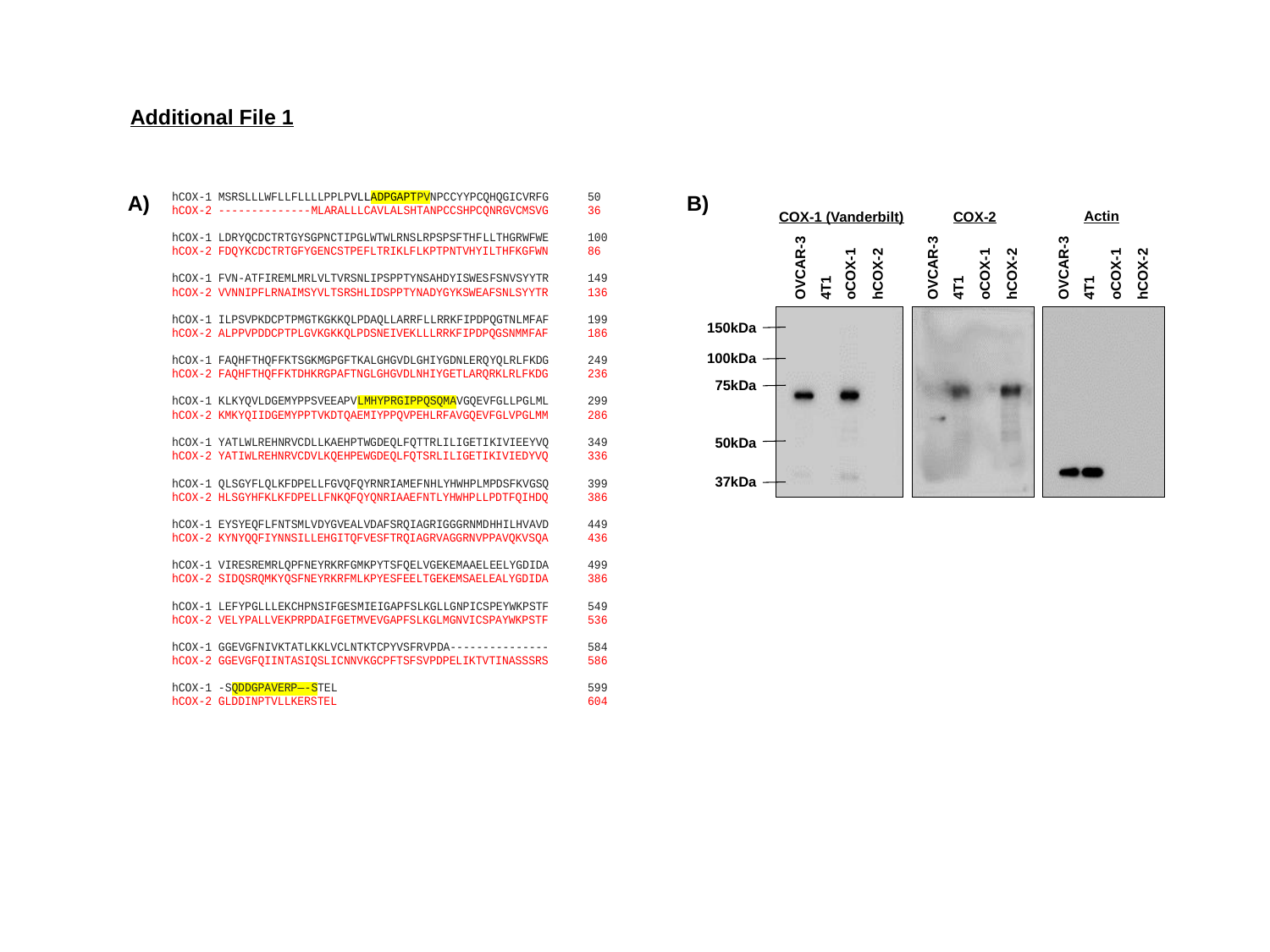

Additional File 1
A)
B)
Actin
COX-1 (Vanderbilt)
COX-2
OVCAR-3
OVCAR-3
OVCAR-3
oCOX-1
hCOX-2
oCOX-1
hCOX-2
oCOX-1
hCOX-2
4T1
4T1
4T1
150kDa
100kDa
75kDa
50kDa
37kDa
