# Supplementary figures and images for: Differential cyclooxygenase expression levels and survival associations in type I and type II ovarian tumors
Source: J Ovarian Res. 2018 Feb 27;11:17. doi: 10.1186/s13048-018-0389-9 (PMC5828488; doi:10.1186/s13048-018-0389-9)

## Slide 1
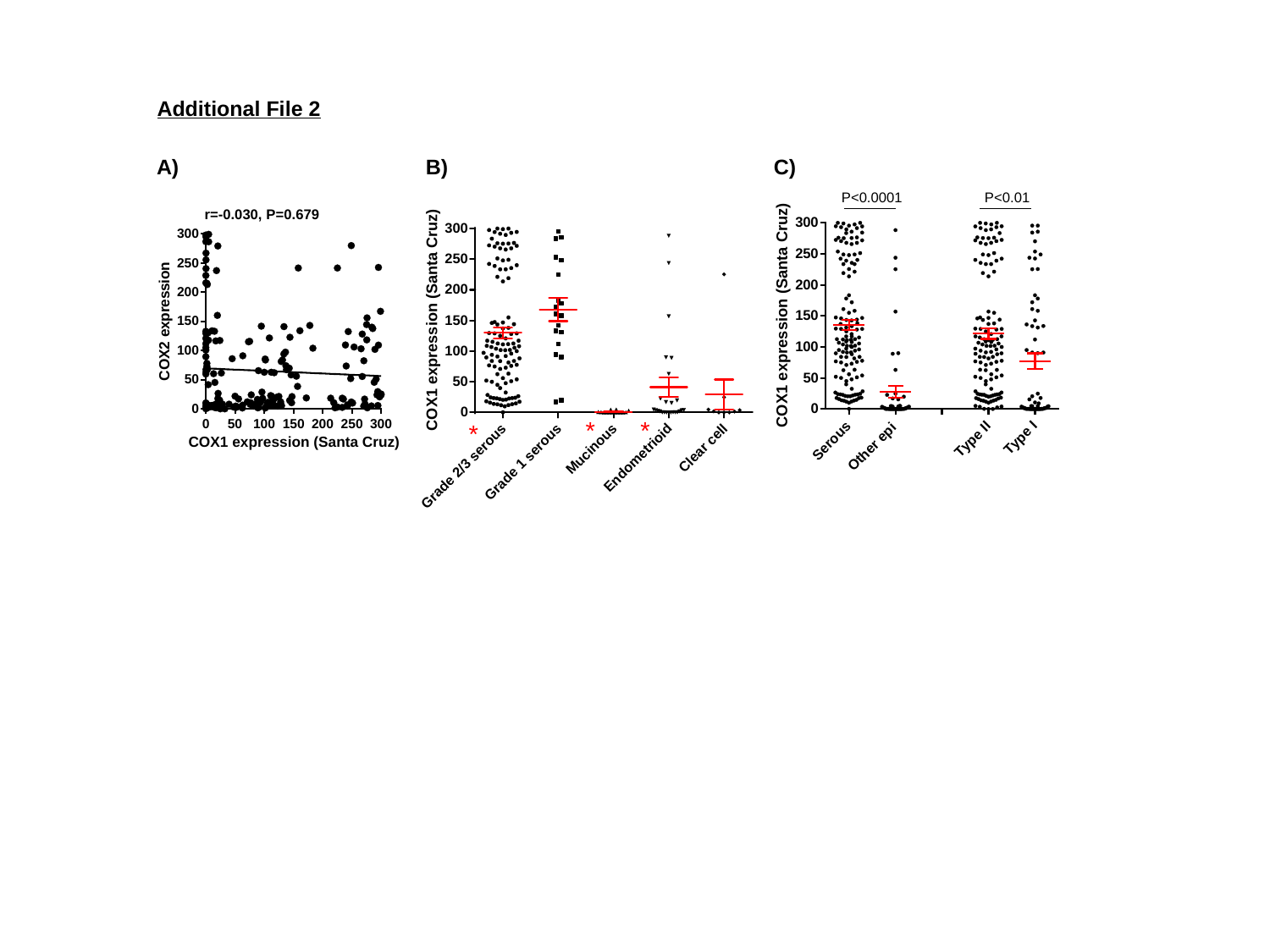

Additional File 2
A)
B)
C)
P<0.0001
P<0.01
*
*
*
r=-0.030, P=0.679

Supplement: Supplementary file 2 — COX-1 expression in ovarian cancer measured by the commercial Santa Cruz antibody (IHC). A) Pearson correlation between IHC expression levels of COX-1 and COX-2. H-scores for COX-1 expression in B) serous, endometrioid, mucinous and clear cell tumors, and in C) serous tumors versus all other epithelial tumors, and type II versus type I tumors. P values were determined by Student’s t-test. (PPTX 377 kb) [file 13048_2018_389_MOESM2_ESM.pptx]

## Slide 1
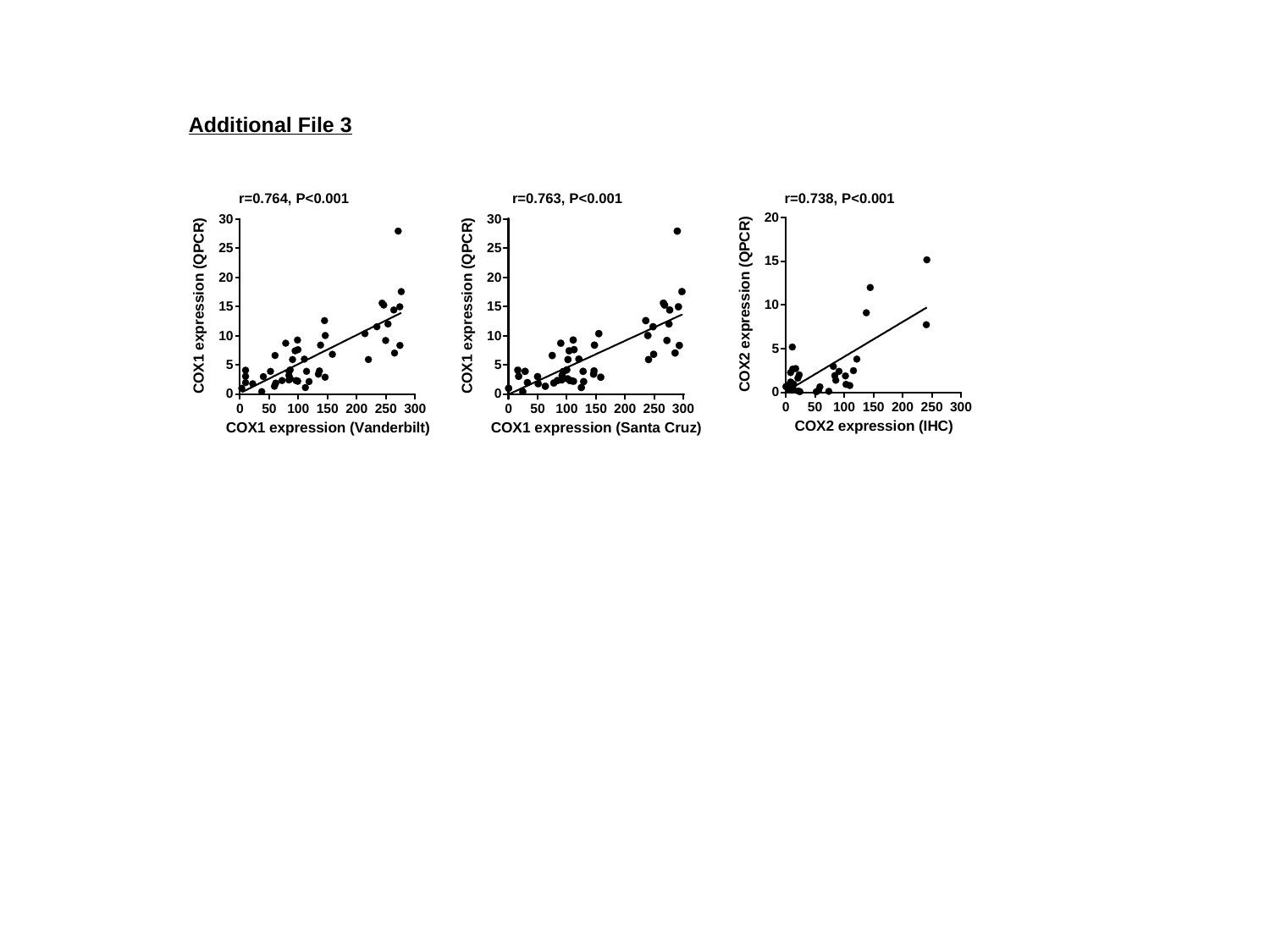

Additional File 3
r=0.763, P<0.001
r=0.738, P<0.001
r=0.764, P<0.001

Supplement: Supplementary file 3 — COX Expression: Correlation between QPCR and IHC. Pearson correlations between COX expression levels measured by quantitative PCR and H-scores from IHC. (PPTX 235 kb) [file 13048_2018_389_MOESM3_ESM.pptx]
